# Surveillance of temporal trends and antimicrobial resistance in nosocomial respiratory pathogens, Switzerland, 2007 to 2024

**DOI:** 10.2807/1560-7917.ES.2026.31.17.2500683

**Published:** 2026-04-30

**Authors:** Christian Bischof, Andreas Kronenberg, Rami Sommerstein, on behalf of the Swiss Centre for Antibiotic Resistance (ANRESIS)

**Affiliations:** 1 Faculty of Healthcare Sciences and Medicine, University of Lucerne, Lucerne, Switzerland; 2 Department of Internal Medicine, Hospital of Nidwalden and Lucerne Cantonal Hospital, Lucerne, Switzerland; 3 Institute for Infectious Diseases (IFIK), University of Bern, Bern, Switzerland; 4 Department of Infectious Diseases, Bern University Hospital, University of Bern, Bern, Switzerland

**Keywords:** Hospital-acquired pneumonia, antimicrobial resistance, Surveillance, Carbapenem-resistant bacteria, Gram-negative pathogens, Switzerland

## Abstract

**BACKGROUND:**

Hospital-acquired pneumonia is a common nosocomial infection, frequently caused by multidrug-resistant organisms. However, no systematic surveillance data exist on respiratory bacteria in Switzerland.

**AIM:**

This laboratory-based surveillance study analysed temporal trends in the incidence and antimicrobial resistance (AMR) of nosocomial respiratory bacteria in Swiss hospitals.

**METHODS:**

A retrospective analysis using data from the Swiss Centre for Antibiotic Resistance from 2007 to 2024 focused on meticillin-resistant *Staphylococcus aureus* (MRSA), third-generation cephalosporin-resistant (3GCR) Enterobacterales, carbapenem-resistant Enterobacterales, carbapenem-resistant *Pseudomonas aeruginosa* and carbapenem-resistant *Acinetobacter* spp. Data were compared across the periods 2007–2015 and 2016–2024. Temporal trends in pathogen incidence and AMR were assessed using Poisson regression models.

**RESULTS:**

We included 46,374 respiratory isolates. For respiratory bacteria incidence, difficult to treat Enterobacterales such as *Serratia* spp. increased significantly in the later vs the earlier period, as reflected by a higher adjusted incidence rate ratio (aIRR: 1.14, 95% confidence interval (CI): 1.06–1.23, p < 0.001). Regarding important pathogen-specific AMR, MRSA (aIRR: 0.78, 95% CI: 0.66–0.91, p = 0.002) and 3GCR Enterobacterales (aIRR: 0.81, 95% CI: 0.75–0.87, p < 0.001) incidence decreased in the later period. In contrast, carbapenem resistance in Enterobacterales (aIRR: 3.27, 95% CI: 2.43–4.46, p < 0.001), *P. aeruginosa* (aIRR: 2.68, 95% CI: 2.33–3.10, p < 0.001) and *Acinetobacter* spp. (aIRR: 1.82, 95% CI: 1.15–2.95, p = 0.01) was higher in the later period.

**CONCLUSIONS:**

While MRSA and 3GCR Enterobacterales incidence declined, carbapenem-resistant Gram-negative bacteria incidence increased, underscoring the need for strengthened surveillance.

**Key public health message**

**What did you want to address in this study and why?**

Hospital-acquired pneumonia is a leading cause of morbidity and mortality in hospitals, but national data on the causative bacteria and their antimicrobial resistance patterns have been lacking in Switzerland. We aimed to analyse long-term trends to detect possible emerging threats.

**What have we learnt from this study?**

Between 2007 and 2024, resistance to methicillin in *Staphylococcus aureus* and to third-generation cephalosporins in Enterobacterales decreased. At the same time, carbapenem resistance rose significantly in Gram-negative bacteria, such as Enterobacterales, *Pseudomonas aeruginosa* and *Acinetobacter* spp.

**What are the implications of your findings for public health?**

The dynamics of detected bacterial pathogens and antimicrobial resistance patterns reflect broader trends observed in hospital settings. The rise in carbapenem-resistant Gram-negative bacteria is particularly concerning, as carbapenems are considered last-line antibiotics, and it underscores the need for strengthened surveillance and infection prevention and control measures.

## Introduction

Hospital-acquired pneumonia (HAP) and ventilator-associated pneumonia (VAP) represent major healthcare-associated infections, contributing substantially to patient morbidity and mortality and healthcare costs. Hospital-acquired pneumonia affects between 5 and 10 patients per 1,000 hospital admissions, while VAP occurs in 10 to 25% of intensive care unit (ICU) patients, accounting for 80% of all ICU-acquired pneumonia cases [1,2]. In recent point prevalence surveys conducted in Switzerland and across Europe, HAP was the second most common healthcare-associated infection in Switzerland and among the most common in Europe, with point prevalences of 1.18% and 1.35%, respectively, among hospitalised patients on the day of the survey [3,4]. Mortality rates range from 13 to 30% for HAP and 20 to 58% for VAP and can exceed 72% in cases involving multidrug-resistant organisms (MDROs) [1,5-10]. The economic burden is substantial. In a recent study conducted in a Spanish tertiary hospital, VAP was associated with additional hospital costs of approximately EUR 20,000 per episode. Similar findings have been reported in other European settings: an analysis from a German teaching hospital estimated that hospital-acquired *Pseudomonas* aeruginosa pneumonia was associated with incremental costs of about EUR 19,000 per case compared with matched hospital controls without P. aeruginosa pneumonia [11,12].

Hospital-acquired pneumonia and VAP are frequently caused by MDROs, particularly meticillin-resistant *Staphylococcus aureus* (MRSA) and carbapenem-resistant Gram-negative bacteria [1,5-10,13-19]. Factors associated with poor clinical outcome such as increased mortality, ICU admission, need for mechanical ventilation, prolonged hospital stay, therapeutic failure, septic shock and multi-organ failure include frequent medical interventions, underlying comorbidities and extended hospital stays, with inappropriate antibiotic therapy as the most significant modifiable risk factor [1,6,7,10,13,20]. Managing HAP and VAP typically requires broad-spectrum antibiotics, potentially accelerating antimicrobial resistance (AMR) development [9,16,20]. While local epidemiology and hospitalisation duration guide empirical antibiotic selection, the relationship between length of stay and AMR pattern remains complex [6,16,21]. Therefore, optimising HAP and VAP management is crucial for reducing unnecessary antibiotic use, limiting resistance development and improving clinical outcome.

Although effective surveillance is fundamental to address AMR, Switzerland currently lacks systematic surveillance data on HAP/VAP bacteria [17,18,22]. This study aims to address this surveillance gap and enhance our understanding of AMR patterns and trends in nosocomial respiratory pathogens across Swiss hospitals. Based on earlier findings, we hypothesised that over the study period, MRSA incidence would decline while carbapenem-resistant Gram-negative bacteria incidence would increase [17-19].

## Methods

### Study design and setting

We conducted a retrospective, laboratory-based surveillance analysis using data from Switzerland's nationwide microbiology laboratory surveillance system. The study period spanned from January 2007 to December 2024 and examined trends in pathogen distribution and AMR patterns among nosocomial respiratory pathogens.

### Data source and representativeness

Data were obtained from the Swiss Centre for Antibiotic Resistance (ANRESIS) database, Switzerland's national surveillance centre for antibiotic resistance and consumption (www.anresis.ch), operated by the Institute for Infectious Diseases at the University of Bern with support from the Swiss Federal Office of Public Health. Swiss laboratories contributing data to ANRESIS perform antimicrobial susceptibility testing following Clinical and Laboratory Standards Institute (CLSI) or European Committee on Antimicrobial Susceptibility Testing (EUCAST) guidelines [23,24]. Laboratories transitioned to EUCAST breakpoints between 2011 and 2013. All participating laboratories maintain Swiss accreditation and participate in external quality programmes, including the United Kingdom National External Quality Assessment Service (UK NEQAS) and/or the Swiss quality control programme at the University of Zurich's Institute for Medical Microbiology. Notification data are currently submitted to ANRESIS from 39 clinical microbiology laboratories distributed across Switzerland, representing ca 90% of annual hospitalisation days across primary to tertiary care facilities. For this study, we included a stable cohort of 24 acute care hospitals, corresponding to ca 29% of Swiss hospitalisation days when referenced against the most recent national hospital statistics (2022) [25]. This hospital cohort and coverage remained consistent throughout the study period, enabling robust longitudinal trend analyses and minimising artefacts from changes in surveillance participation.

### Study population and isolate selection

We considered laboratory surveillance data from respiratory samples - sputum, tracheobronchial secretions (TBS) and bronchoalveolar lavage (BAL) - obtained from inpatients at Swiss acute care hospitals and processed in participating clinical microbiology laboratories. As ANRESIS data coverage was limited in the early years and became consistent from 2007 onward, with continuous participation of the same hospitals, the study period began in 2007.

**Figure 1:**
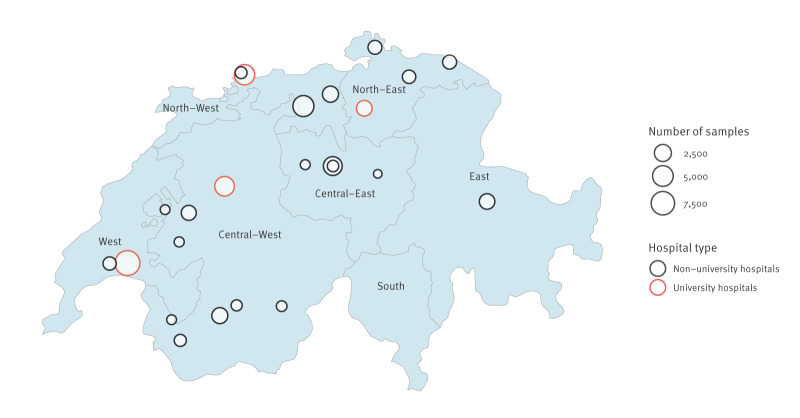
Geographic distribution of included isolates and hospital types across geographic regions, Switzerland, 2007–2024 (n = 24)

We restricted inclusion to hospitals providing information on the day of hospital admission as well as on the sampling day. To ensure data quality and stable longitudinal coverage, we included only hospitals that continuously submitted an average of at least 10 respiratory samples per year throughout the study period, resulting in data from the same 24 acute care hospitals (details on participating hospitals are provided in Supplementary Table S1). All respiratory isolates from participating hospitals are submitted to ANRESIS, with no sampling or selective reporting at the hospital level. The geographical distribution and care level allocation of secondary and tertiary hospitals, along with their provided sample sizes, are illustrated in a geographical map of Switzerland (Figure 1). To prevent duplicate sampling, we included only the first isolate per patient-microorganism combination. While multiple species from a single patient were eligible, only the initial isolate of each species was analysed.

**Figure 2:**
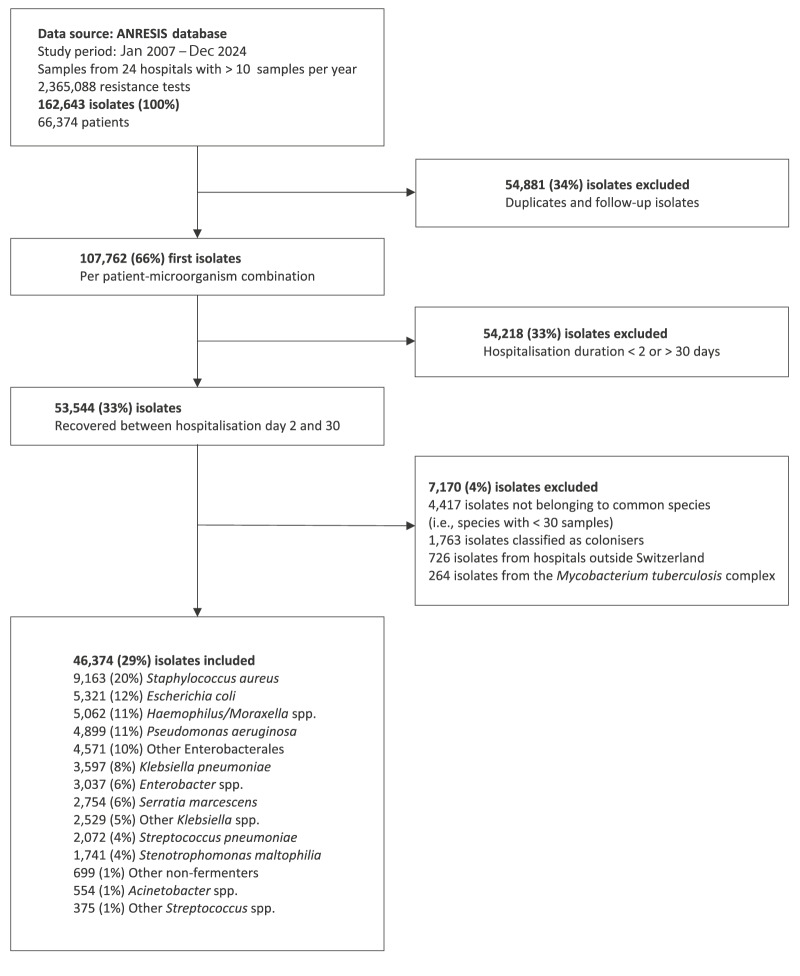
Flowchart illustrating inclusion and exclusion criteria for respiratory isolates from acute care hospitals submitted to the Swiss Centre for Antibiotic Resistance (ANRESIS) database, Switzerland, 2007–2024

To focus on nosocomial infections, we excluded samples collected within 2 days of admission (days 0–1). As ANRESIS does not capture clinical diagnoses, references to HAP and VAP represent microbiological proxies based solely on specimen timing beyond this 48-hour threshold. To avoid extreme outliers reflecting long-term hospital stays and chronic care pathways not representative of an acute care setting, we additionally excluded samples collected after 30 days of hospitalisation. This ensured comparability of patient populations and maintained a focus on acute nosocomial infections. Additional exclusion criteria comprised species with fewer than 30 samples (i.e. not belonging to the common species associated with HAP) and colonising organisms (including *Candida albicans*, coagulase-negative *Staphylococcus*, *Enterococcus* spp., *Corynebacterium* spp.). To focus on typical HAP/VAP bacterial pathogens, we also excluded *Mycobacterium tuberculosis* complex and *Aspergillus fumigatus* isolates (Figure 2).

### Definition of antimicrobial resistance groups

We defined resistance according to the 2019 EUCAST standards, including the ‘susceptible, increased exposure’ category into the susceptible group. We categorised the following specific resistance groups: (i) MRSA, defined as *S. aureus* resistant to oxacillin and/or other indicators of meticillin resistance, such as cefoxitin (with optional molecular confirmation of *mecA* or PBP2a in some laboratories), (ii) third-generation cephalosporin-resistant (3GCR) Enterobacterales, defined as resistance to ceftriaxone, ceftazidime, cefpodoxime, cefotaxime or cefixime, (iii) carbapenem-resistant Enterobacterales (CRE), (iv) carbapenem-resistant *P. aeruginosa* (CRPA), and (v) carbapenem-resistant *Acinetobacter* spp. (CRA), with each of the latter three defined as resistant to at least one of the following carbapenems: imipenem, meropenem or ertapenem. All susceptibility testing was primarily culture-based. Resistance was considered present if at least one antimicrobial agent within the respective antibiotic group was reported as resistant.

### Variables and outcomes

Available epidemiological data included patient identifiers, admission and sampling dates, patient sex and age group (younger than 60 years vs 60 years and older), hospital type, hospital department, geographic region and recovery method. Covariates were categorised as host factors (sex, age group), environmental factors (geographic region, hospital type, hospital department) and recovery factors (recovery method, sampling day after admission). Geographic regions were defined according to the classification used in the Swiss ANRESIS database (Central-East, Central-West, East, North-East, North-West, West; Figure 1). Clinical data, including antibiotic prescriptions, were unavailable. Outcome one was the incidence of respiratory pathogens at the species level. Outcome two was the AMR of selected pathogens according to the predefined resistance groups.

### Statistical analysis

Temporal changes in pathogen distribution and AMR were analysed using the same modelling approach. For both outcomes, binary variables were defined: pathogen of interest vs all other pathogens, and resistant vs non-resistant within predefined resistance groups. We applied Poisson regression to estimate crude and adjusted incidence rate ratios (IRR, aIRR) with 95% confidence intervals (CI), adjusting for the predefined covariates. For parsimony reasons, we compared the two periods 2007–2015 and 2016–2024. Categorical variables were compared between the two periods using chi-square tests. Sensitivity analyses for both pathogen distribution and AMR reestimated temporal trends using the calendar year as a continuous variable. For pathogen distribution, we applied a heteroskedasticity-consistent (robust) variance estimator to address numerical instability when modelling the calendar year as a continuous variable. This robustness requirement was the sole reason for deviating from the standard variance estimation used in all main analyses and all resistance models. For AMR, resistance rates (RR) were additionally reported as the proportion of resistant isolates among tested isolates. Statistical significance was set at p < 0.05. Analyses were performed using R version 4.3.0 [26].

## Results

### Sample characteristics

From 162,643 respiratory isolates collected from 66,374 patients, 46,374 (29%) met the inclusion criteria (Figure 2). *S. aureus* constituted the largest group, followed by *Escherichia coli*, *Haemophilus*/*Moraxella* spp., *P. aeruginosa* and other Enterobacterales, collectively representing 62.6% of samples.

Comparing 2007–2015 with 2016–2024, the proportion of male patients decreased marginally, while patients aged 60 years or older increased significantly. Regional distribution showed modest but significant shifts, with increased representation from the Eastern and Western regions and decreased sampling from the Central regions. University hospital and ICU samples decreased. Recovery methods showed the most pronounced changes, with increased sputum collection, decreased TBS sampling and stable BAL rates. Sample timing remained consistent, with most collections occurring between days 2–4 of hospitalisation (Table 1).

**Table 1:** Epidemiological characteristics of nosocomial respiratory isolates submitted to the Swiss Centre for Antibiotic Resistance (ANRESIS) database, stratified by time periods, Switzerland, 2007–2015 and 2016–2024

Variable	2007–2015		2016–2024		p value
	n	%	n	%	
Number of isolates (n = 46,374)					
Total	21,997	47.4	24,377	52.6	NA
Sex					
Male	15,514	70.5	16,923	69.4	0.01
Female	6,463	29.5	7,454	30.6	
Age group					
Age ≥ 60 years	13,958	63.5	16,610	68.1	< 0.001
Age < 60 years	8,039	36.5	7,767	31.9	
Geographic region					
Central-East	2,300	10.5	2,233	9.2	< 0.001
Central-West	4,741	21.6	5,180	21.2	
East	791	3.6	1,114	4.6	
North-East	2,358	10.7	2,801	11.5	
North-West	6,603	30.0	7,046	28.9	
West	5,204	23.7	6,003	24.6	
Hospital type					
University hospital	10,821	49.2	11,250	46.2	< 0.001
Non-university hospital	11,176	50.8	13,127	53.8	
Hospital department					
Non-intensive care unit	11,039	50.2	13,276	54.5	< 0.001
Intensive care unit	10,958	49.8	11,101	45.5	
Recovery method					
Sputum	6,666	30.3	9,253	38.0	< 0.001
Tracheobronchial secretion	14,130	64.2	13,734	56.3	
Bronchoalveolar lavage	1,201	5.5	1,390	5.7	
Sampling day after admission					
Day 2–4	8,769	39.9	9,953	40.8	0.09
Day 5–10	7,248	32.9	7,950	32.6	
Day 11–30	5,980	27.2	6,474	26.6	

NA: not applicable.

Data are presented as number (%) for categorical variables. P values are based on chi-square tests comparing the two time periods (2007–2015 vs 2016–2024). Statistical significance was set at p < 0.05.

### Pathogen distribution trends

**Figure 3:**
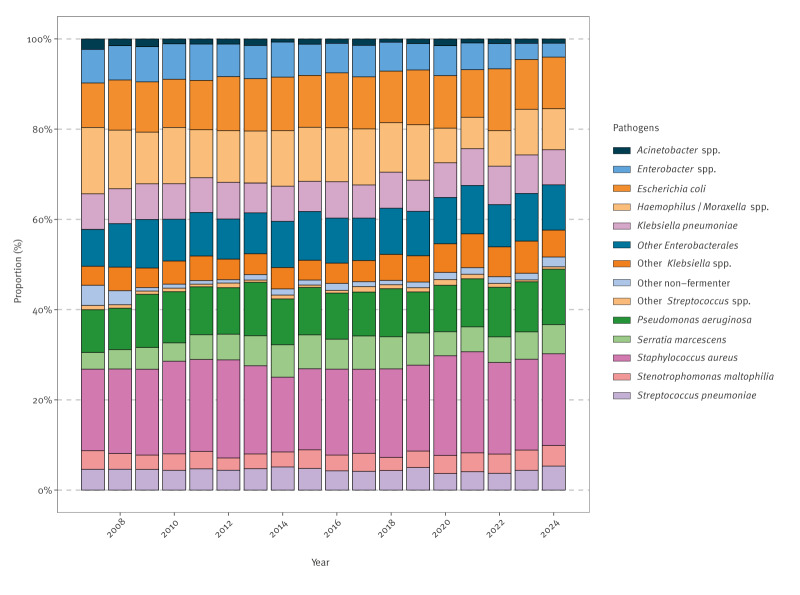
Annual distribution of nosocomial respiratory pathogens submitted to the Swiss Centre for Antibiotic Resistance (ANRESIS) database, Switzerland, 2007–2024

The annual distribution of pathogens over the study period is shown in Figure 3 (with corresponding absolute counts presented in Supplementary Table S2). Pathogen distribution was assessed using modelled temporal trends expressed as IRR and aIRR. Temporal analysis comparing the periods 2007–2015 and 2016–2024 (detailed results in Supplementary Table S3) revealed significant increases in other *Klebsiella* spp. (aIRR: 1.29, 95% CI: 1.19–1.39, p < 0.001), other *Streptococcus* spp., *Serratia marcescens* (aIRR: 1.14, 95% CI: 1.06–1.23, p < 0.001), *Stenotrophomonas maltophilia* and *S. aureus*. Significant decreases were observed in *Enterobacter* spp. (aIRR: 0.75, 95% CI: 0.69–0.80, p < 0.001), *Acinetobacter* spp., *Haemophilus*/*Moraxella* spp. and *Streptococcus pneumoniae*. *E. coli*, *P. aeruginosa*, other Enterobacterales, *K. pneumoniae* and other non-fermenters showed no statistically significant changes. Sensitivity analysis using annual IRR/aIRR confirmed these trends (detailed results in Supplementary Table S4).

### Antimicrobial resistance trends

**Figure 4:**
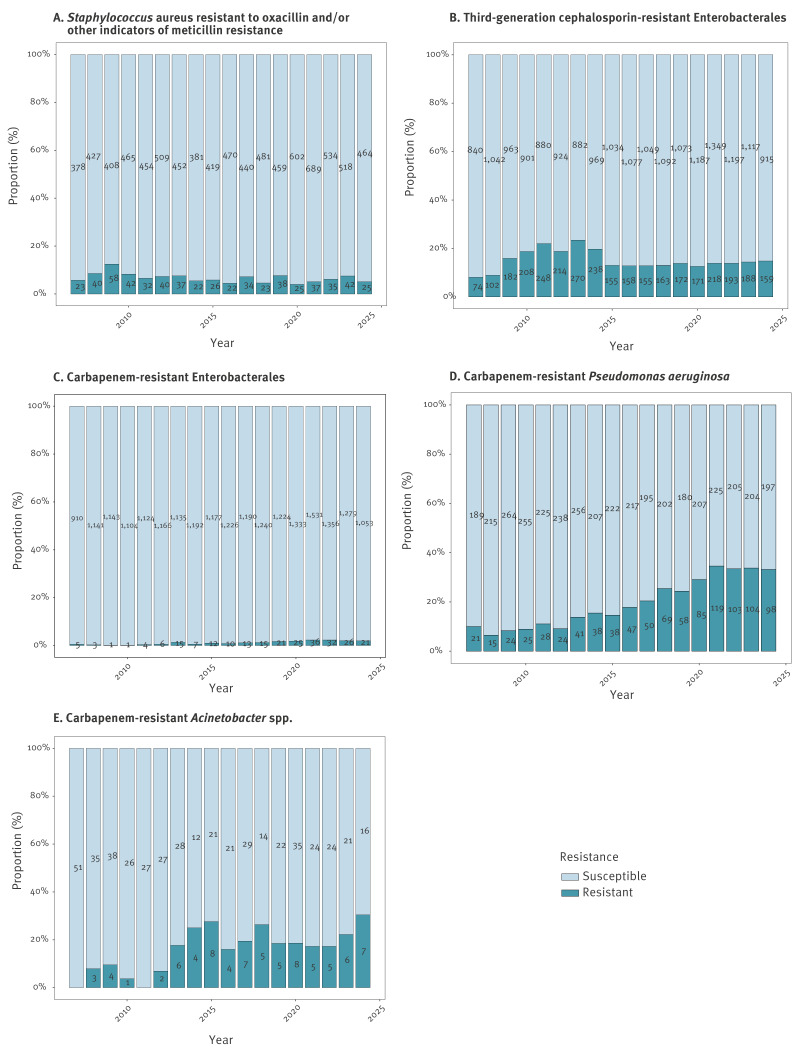
Temporal trends in resistance rates across resistance groups in nosocomial respiratory isolates submitted to the Swiss Centre for Antibiotic Resistance (ANRESIS) database, Switzerland, 2007–2024

Antimicrobial resistance of selected pathogens was analysed using RR and modelled temporal trends expressed as IRR and aIRR. The analysis of RR revealed distinct trends across different resistance groups (Figure 4, Table 2). Meticillin resistance within *S. aureus* was lower in 2016–2024 (5.7%) compared with 2007–2015 (7.6%). A similar trend was observed for third-generation cephalosporin resistance within Enterobacterales, which decreased from 16.7% to 13.6%. In the same periods, carbapenem resistance increased across multiple pathogens: within Enterobacterales, resistance rose from 0.5% to 1.7%, in *P. aeruginosa* from 10.9% to 28.6%, and in *Acinetobacter* spp. from 9.6% to 20.2%. Covariate-adjusted temporal resistance patterns (Table 2) showed declining MRSA (aIRR: 0.78, 95% CI: 0.66–0.91, p = 0.002) and 3GCR Enterobacterales (aIRR: 0.81, 95% CI: 0.75–0.87, p < 0.001) between 2016 and 2024 compared with 2007–2015. Conversely, carbapenem resistance increased in Enterobacterales (aIRR: 3.27, 95% CI: 2.43–4.46, p < 0.001), *P. aeruginosa* (aIRR: 2.68, 95% CI: 2.33–3.10, p < 0.001), and *Acinetobacter* spp. (aIRR: 1.82, 95% CI: 1.15–2.95, p = 0.01). In summary, this demonstrates a significant increase in carbapenem resistance for Enterobacterales, *P. aeruginosa*, and *Acinetobacter* spp., while trends for MRSA and 3GCR Enterobacterales were declining. Sensitivity analysis using annual IRR/aIRR confirmed these trends (detailed results in Supplementary Table S5).

**Table 2:** Resistance rates, unadjusted and adjusted incidence rate ratios across resistance groups in nosocomial respiratory isolates submitted to the Swiss Centre for Antibiotic Resistance (ANRESIS) database, Switzerland, 2007–2015 vs 2016–2024

Resistance group	Resistance rates				Univariate analysis			Multivariate analysis ^a^		
	RR 2007–2015		RR 2016–2024		IRR 2016–2024 (Reference: 2007–2015)			aIRR 2016–2024 (Reference: 2007–2015)		
	n/n	%	n/n	%	IRR	95% CI	p value	aIRR	95% CI	p value
Meticillin resistance within tested *Staphylococcus aureus* (601/9,151, 6.6%)	320/4,213	7.6	281/4,938	5.7	0.75	0.64–0.88	< 0.001	0.78	0.66–0.91	0.002
Third-generation cephalosporin resistance within tested Enterobacterales (3,268/21,759, 15.0%)	1,691/10,126	16.7	1,577/11,633	13.6	0.81	0.76–0.87	< 0.001	0.81	0.75–0.87	< 0.001
Carbapenem resistance within tested Enterobacterales (253/21,777, 1.2%)	54/10,146	0.5	199/11,631	1.7	3.21	2.40–4.38	< 0.001	3.27	2.43–4.46	< 0.001
Carbapenem resistance within tested *Pseudomonas aeruginosa* (987/4,890, 20.2%)	254/2,325	10.9	733/2,565	28.6	2.62	2.27–3.02	< 0.001	2.68	2.33–3.10	< 0.001
Carbapenem resistance within tested *Acinetobacter* spp. (80/551, 14.5%)	28/293	9.6	52/258	20.2	2.11	1.34–3.38	0.002	1.82	1.15–2.95	0.010

aIRR: adjusted incidence rate ratio; IRR: incidence rate ratio; RR: resistance rates.

^a^ Adjusted for covariates: sex, age group, geographic region, hospital type, hospital department, recovery method, sampling day after admission (for full analysis, see Supplementary Table S6).

Covariate analysis revealed several statistically significant patterns across resistance groups (full model results of adjusted IRR across all resistance groups are shown in Supplementary Table S6): Male sex was associated with lower aIRR for 3GCR Enterobacterales (aIRR: 0.91, 95% CI: 0.85–0.98, p = 0.02). Age 60 years or older correlated with increased aIRR for MRSA (aIRR: 1.54, 95% CI: 1.29–1.85, p < 0.001) and 3GCR Enterobacterales (aIRR: 1.23, 95% CI: 1.13–1.34, p < 0.001). The West region showed the highest aIRR for MRSA (aIRR: 4.68, 95% CI: 3.02–7.46, p < 0.001), while the East region had elevated aIRR for CRA (aIRR: 4.92, 95% CI: 1.55–16.89, p = 0.007). Hospital type showed no significant impact. Non-ICU settings had lower aIRR for 3GCR Enterobacterales (aIRR: 0.87, 95% CI: 0.80–0.94, p < 0.001) but higher aIRR for CRPA (aIRR: 1.19, 95% CI: 1.02–1.38, p = 0.02). Tracheobronchial secretion samples showed lower aIRR for MRSA (aIRR: 0.70, 95% CI: 0.51–0.99, p = 0.04) compared to BAL. Sampling at later time points after admission was generally associated with higher resistance. However, for CRA, sampling at day 11–30 was associated with lower aIRR (aIRR: 0.44, 95% CI: 0.25–0.76, p = 0.003).

## Discussion

Our laboratory-based analysis of national surveillance data examined temporal trends in the distribution and resistance patterns of nosocomial respiratory pathogens in Swiss hospitals. We observed a significant increase in challenging Enterobacterales, particularly *Serratia* spp. Regarding resistance trends, MRSA and 3GCR Enterobacterales declined, whereas carbapenem resistance significantly increased in Enterobacterales, *P. aeruginosa* and *Acinetobacter* spp.

The MRSA decline observed in our study reflects broader AMR trends in Switzerland. This decline was most pronounced in the West region of Switzerland, potentially attributable to stricter infection control measures implemented following a past MRSA epidemic in that region [27]. Nevertheless, our study found that the West region still maintains the highest aIRR for MRSA (aIRR: 4.68, 95% CI: 3.02–7.46, p < 0.001), indicating a comparatively high regional burden despite the overall decline. Of note, because our results are restricted to respiratory samples, direct comparisons with surveillance data based on invasive samples (blood culture, cerebrospinal fluid) are limited. The same applies to all pathogens discussed below.

Regarding 3GCR Enterobacterales, we observed a temporal shift. While a 2004–2011 surveillance study reported a significant increase in extended-spectrum cephalosporin-resistant *E. coli* and *K. pneumoniae* in Switzerland [28], our 2016–2024 data demonstrate a decreasing trend in 3GCR Enterobacterales overall.

Our findings regarding CRE align with recent Swiss ICU data, which show an increase in CRE among respiratory isolates, particularly among *Enterobacter* spp., with RR reaching 5% in 2018 [17]. Carbapenem-resistant Enterobacterales were more frequently detected in Eastern Switzerland, consistent with our observation of the highest regional proportions. This trend is supported by national surveillance data reporting that carbapenemase-producing Enterobacterales cases in Switzerland tripled between 2013 and 2018.

Recent national surveillance data reported stable carbapenem RR of ca 9% in *P. aeruginosa* bloodstream infections between 2010 and 2022, based on resistance to imipenem and/or meropenem [29]. Similarly, Swiss ICU data reported stable CRPA rates of ca 27% between 2009 and 2018 [17]. In contrast to these findings, our respiratory isolate data reveal a marked increase in CRPA over the same period, suggesting potentially higher selective pressure or differing resistance dynamics in non-invasive settings. Notably, a clear increase in CRPA disease burden was observed in Switzerland, with disability-adjusted life years approximately doubling from ca 500 in 2010 to ca 1,000 in 2019, predominantly in university hospitals [18]. In our dataset, 28.6% of *P. aeruginosa* isolates were carbapenem-resistant between 2016 and 2024, with increasing rates over time. This places Switzerland at the lower end of resistance levels reported from neighbouring countries.

A similar pattern emerged with *Acinetobacter* spp. We found high carbapenem RR (20.2%) during 2016–2024, with a clear upward trend. Current national surveillance data confirm an increase with CRA rates reaching 13% in 2023 [30]. Consistent with our findings, the highest RR were observed in the East and North-East region of Switzerland.

In our study, RR across most antimicrobial groups increased significantly with longer hospitalisation duration, consistent with the established association between prolonged hospital stays and AMR. Interestingly, this trend did not apply to CRA, where RR significantly decreased with longer hospitalisation. While the reasons remain unclear, one explanation may involve the identification of screening isolates from transferred or readmitted patients. Notably, this complexity aligns with previous findings demonstrating that resistance patterns in respiratory isolates from HAP do not follow the simplistic early vs late dichotomy proposed by current pneumonia treatment guidelines, but rather exhibit a complex, nonlinear relationship shaped by various host and environmental factors [21].

Another noteworthy finding in our study was that sputum and TBS samples consistently showed lower aIRR for drug resistance compared with BAL. While often not significant, this difference reached significance for MRSA in TBS. This observation warrants consideration, as higher RR of MRSA in TBS have been described as a consequence of frequent colonisation and biofilm formation on airway devices [31]. Our results may reflect sampling selection bias. Bronchoalveolar lavage is primarily performed in critically ill patients with severe or treatment-refractory infections where MDRO detection likelihood is inherently higher. In a broader European context, respiratory tract infections represent one of the most common healthcare-associated infections in acute care hospitals, underlining the importance of monitoring AMR in respiratory pathogens. Direct comparisons should be interpreted with caution, as European surveillance systems such as European Antimicrobial Resistance Surveillance Network (EARS-Net) are based on invasive isolates, whereas our analysis focuses on respiratory samples. Furthermore, we compared pooled estimates from 2016–2024 with population-weighted European Union/European Economic Area (EU/EEA) means for 2024 and contrasted temporal trends in our dataset over 2016–2024 with European trends reported for 2020–2024. Within these limitations, the following comparisons can be made: declining MRSA and 3GCR Enterobacterales rates in our study are broadly consistent with European data. Carbapenem-resistant Enterobacterales rates in our cohort (1.7%) fall within the wide interspecies variability reported in Europe, where mean rates of carbapenem resistance are 0.3% for *E. coli* and 11.3% for *K. pneumoniae* (country ranges: 0.0–2.5% and 0.0–67.6%, respectively), with increasing trends observed both in our dataset and across Europe. Carbapenem-resistant *P. aeruginosa* rates were higher in our study (28.6%) than the EU/EEA mean (15.9%, country range: 1.5–53.4%), with increasing trends in our data compared to a decreasing trend in Europe. Carbapenem-resistant *Acinetobacter* spp. rates were lower in our dataset (20.2%) than the EU/EEA mean (31.6%, country range: 0.0–94.1%), with increasing trends in our data and decreasing trends in Europe. The highest resistance levels in Europe were consistently reported from southern and eastern regions, highlighting substantial geographic heterogeneity [32].

Effective AMR prevention strategies are critical, with inappropriate antibiotic use being the primary modifiable risk factor. European ICU surveillance data revealed that 30% of patients with *P. aeruginosa* infections received inadequate empirical therapy [6]. Prior use of carbapenems (odds ratio (OR): 4.36) and piperacillin-tazobactam (OR: 2.64) has been linked to CRPA emergence [33]. In Switzerland, institutional antibiotic use was associated with CRE emergence, particularly driven by AmpC-derepressed *Enterobacter* spp. [17].

Timely initiation of appropriate empirical therapy is essential, with regimens guided by local epidemiology, patient-specific risk factors, illness severity and duration of hospitalisation. Antimicrobial stewardship programmes are vital for preventing overuse and preserving antibiotic efficacy by promoting short treatment durations, early de-escalation and restricted carbapenem use [6].

Our study has several limitations. First, microbiological data were limited. We did not analyse resistance mechanisms or perform strain typing, which did not allow stratification by resistance genes. Moreover, the isolate collection was influenced by clinicians’ culturing preferences, and some hospitals may have submitted routine surveillance cultures. Furthermore, differences in testing methods between laboratories (CLSI or EUCAST), along with standard changes during the study period (most of the participating laboratories switched from CLSI to EUCAST breakpoints between 2011 and 2013), may have affected data comparability. And although we attempted to exclude potential colonisers, some residual colonisation cannot be entirely ruled out. Second, differences in epidemiological characteristics between the two study periods may have introduced bias. Given the large sample size, even small shifts reached statistical significance, resulting in hyperinflated p values. As crude IRR and aIRR were highly consistent, we consider substantial bias unlikely. However, residual confounding cannot be fully excluded. Third, clinical data were incomplete. Clinical diagnoses such as HAP or VAP were not available and these categories were inferred based on specimen timing. Prior antibiotic use was not documented, neither before nor during hospitalisation, limiting our ability to assess its direct impact on resistance patterns. As clinical diagnoses and sampling indications are not captured in the ANRESIS database, a clear distinction between surveillance and clinical cultures or between colonisation and infection cannot be achieved. Fourth, representativeness of the dataset was limited. The analysed hospital cohort represents ca 29% of Swiss hospitalisation days, which may restrict national generalisability. Furthermore, geographical representation was not entirely balanced. While hospitals were widely distributed across Switzerland, data from the South region were missing and regional differences in sample size and microbiological testing intensity may have introduced bias, with the East region being underrepresented. Finally, we did not investigate the potential impact of the COVID-19 pandemic on our findings.

We believe that our results are transferable to other healthcare systems with similar resources, especially in Europe. Generalisability to countries with limited resources or higher RR may be less, but our methodology lends itself to similar evaluations in these not directly comparable systems.

## Conclusion

This study provides long-term insight into specific resistance patterns in respiratory isolates, offering a complementary perspective to the usual focus on invasive isolates in AMR dynamics. Our hypothesis that the incidence of MRSA would decline while carbapenem-resistant Gram-negative bacteria would increase was confirmed. The extent of resistance development is critical and underscores the need for strengthened surveillance and effective antibiotic stewardship. Incorporating current surveillance data when selecting empirical therapy is essential for optimal patient outcomes.

## Data Availability

No sequence data were generated in this study. Antibiotic susceptibility data from the surveillance are available from the corresponding author on reasonable request.

## Supplementary Data

654000 Supplementary Material
