# Supplementary material for: Surveillance of temporal trends and antimicrobial resistance in nosocomial respiratory pathogens, Switzerland, 2007 to 2024
Source: Euro Surveill. 2026 Apr 30;31(17):2500683. doi: 10.2807/1560-7917.ES.2026.31.17.2500683 (PMC13135151; doi:10.2807/1560-7917.ES.2026.31.17.2500683)
Supplement: Supplementary Material [file 25-00683_BISCHOF_Supplement.pdf]

Supplementary Material

This supplementary material is hosted by *Eurosurveillance* as supporting information alongside the article *Surveillance of temporal trends and antimicrobial resistance in nosocomial respiratory pathogens, Switzerland, 2007 to 2024*, on behalf of the authors, who remain responsible for the accuracy and appropriateness of the content. The same standards for ethics, copyright, attributions and permissions as for the article apply. Supplements are not edited by *Eurosurveillance* and the journal is not responsible for the maintenance of any links or email addresses provided therein.

Supplementary Table S1. List of hospitals providing respiratory samples (included isolates only)

Number	City	Hospital name	Sample size
1	Aarau	Kantonsspital Aarau	5,485
2	Baden	Kantonsspital Baden	2,257
3	Basel	Universitätsspital Basel	5,442
4	Basel	REHAB Basel	477
5	Bern	Universitätsspital Bern	4,544
6	Chur	Kantonsspital Graubünden	1,897
7	Frauenfeld	Kantonsspital Thurgau	977
8	Fribourg	Hôpital Fribourgeois	1,554
9	Lausanne	Centre Hospitalier Universitaire Vaudois (CHUV)	10,171
10	Luzern	Kantonsspital Luzern	3,948
11	Martigny	Centre Hospitalier du Valais Romand	632
12	Morges	Ensemble Hospitalier de la Côte	929
13	Münsterlingen	Kantonsspital Thurgau	1,238
14	Payerne	Hôpital Intercantonal de la Broye	107
15	Riaz	Hôpital Fribourgeois	135
16	Schaffhausen	Kantonsspital Schaffhausen	1,030
17	Schwyz	Kantonsspital Schwyz	63
18	Sierre	Centre Hospitalier du Valais Romand	320
19	Sion	Centre Hospitalier du Valais Romand	2,328
20	St. Maurice	Clinique Saint-Amé	118
21	Sursee	Kantonsspital Luzern	391
22	Visp	Spitalzentrum Oberwallis	287
23	Wolhusen	Kantonsspital Luzern	130
24	Zürich	Universitätsspital Zürich	1,914

Supplementary Table S2. Annual distribution of nosocomial respiratory pathogens, 2007–2024

Period 2007–2015																		
	2007		2008		2009		2010		2011		2012		2013		2014		2015	
	n	%	n	%	n	%	n	%	n	%	n	%	n	%	n	%	n	%
Total	2,219	100	2,507	100	2,448	100	2,472	100	2,384	100	2,539	100	2,508	100	2,433	100	2,487	100
Staphylococcus aureus	401	18.1	470	18.7	466	19.0	507	20.5	486	20.4	552	21.7	490	19.5	403	16.6	447	18.0
Escherichia coli	218	9.8	279	11.1	273	11.2	264	10.7	261	10.9	304	12.0	291	11.6	289	11.9	286	11.5
Haemophilus/*Moraxella* spp.	327	14.7	325	13.0	280	11.4	308	12.5	253	10.6	291	11.5	289	11.5	299	12.3	298	12.0
Pseudomonas aeruginosa	210	9.5	230	9.2	288	11.8	280	11.3	253	10.6	262	10.3	297	11.8	247	10.2	261	10.5
Other Enterobacterales	181	8.2	242	9.7	263	10.7	229	9.3	230	9.6	226	8.9	227	9.1	249	10.2	269	10.8
Klebsiella pneumoniae	175	7.9	194	7.7	194	7.7	195	7.9	184	7.7	206	8.1	166	6.6	190	7.8	166	6.7
Enterobacter spp.	166	7.5	190	7.6	191	7.8	194	7.8	192	8.1	183	7.2	185	7.4	189	7.8	172	6.9
Serratia marcescens	82	3.7	108	4.3	118	4.8	101	4.1	131	5.5	144	5.7	167	6.7	175	7.2	187	7.5
Other *Klebsiella* spp.	94	4.2	131	5.2	106	4.3	127	5.1	130	5.5	116	4.6	116	4.6	115	4.7	109	4.4
Streptococcus pneumoniae	102	4.6	116	4.6	112	4.6	108	4.4	112	4.7	112	4.4	119	4.7	125	5.1	119	4.8
Stenotrophomonas maltophilia	92	4.1	87	3.5	78	3.2	91	3.7	92	3.6	69	2.7	82	3.3	81	3.3	103	4.1
Other non-fermentes	99	4.5	78	3.1	19	0.8	22	0.9	19	0.8	19	0.7	30	1.2	33	1.4	28	1.1
Acinetobacter spp.	51	2.3	38	1.5	42	1.7	27	1.1	27	1.1	29	1.1	36	1.4	17	0.7	29	1.2
Other *Streptococcus* spp.	21	0.9	19	0.8	18	0.7	19	0.8	14	0.6	26	1.0	13	0.5	21	0.9	13	0.5

Period 2016–2024																		
	2016		2017		2018		2019		2020		2021		2022		2023		2024	
	n	%	n	%	n	%	n	%	n	%	n	%	n	%	n	%	n	%
Total	2,581	100	2,543	100	2,567	100	2,613	100	2,837	100	3,240	100	2,808	100	2,786	100	2,402	100
Staphylococcus aureus	492	19.1	474	18.6	504	19.6	498	19.1	627	22.1	726	22.4	570	20.3	561	20.1	489	20.4
Escherichia coli	314	12.1	293	11.5	293	11.4	317	12.1	330	11.6	342	10.6	385	13.7	307	11.0	275	11.4
Haemophilus/*Moraxella* spp.	309	12.0	316	12.4	282	11.0	321	12.3	218	7.7	225	6.9	221	7.9	282	10.1	218	9.1
Pseudomonas aeruginosa	264	10.2	247	9.9	273	10.8	238	9.0	292	9.7	345	11.4	309	10.3	309	11.1	295	12.3
Other Enterobacterales	258	10.0	239	9.4	263	10.2	257	9.8	291	10.3	348	10.7	263	9.4	295	10.6	241	10.0
Klebsiella pneumoniae	208	8.1	187	7.4	205	8.0	181	6.9	218	7.7	264	8.1	239	8.5	238	8.5	187	7.8
Enterobacter spp.	169	6.5	178	7.0	164	6.4	153	5.9	188	6.6	192	5.9	157	5.6	100	3.6	74	3.1
Serratia marcescens	171	6.6	188	7.4	183	7.1	186	7.1	151	5.3	178	5.5	160	5.7	169	6.1	155	6.5
Other *Klebsiella* spp.	116	4.5	119	4.7	148	5.8	152	5.8	180	6.3	243	7.5	186	6.6	198	7.1	143	6.0
Streptococcus pneumoniae	110	4.3	106	4.2	111	4.3	130	5.0	104	3.7	132	4.1	104	3.7	122	4.4	128	5.3
Stenotrophomonas maltophilia	90	3.5	101	4.0	75	2.9	96	3.7	114	4.0	136	4.2	120	4.3	125	4.5	109	4.5
Other Non−Fermenter	40	1.5	28	1.1	24	0.9	33	1.3	45	1.6	47	1.5	42	1.5	41	1.5	52	2.2
Acinetobacter spp.	25	1.0	36	1.4	19	0.7	27	1.0	43	1.5	29	0.9	29	1.0	27	1.0	23	1.0
Other *Streptococcus* spp.	15	0.6	31	1.2	23	0.9	24	0.9	36	1.3	33	1.0	24	0.9	12	0.4	13	0.5

Supplementary Table S3. Pathogen distribution trends, unadjusted and adjusted incidence rate ratios in nosocomial respiratory isolates, 2007–2015 vs. 2016–2024

Overview pathogen distribution		Univariate Analysis			Multivariate Analysis [1]		
Microorganism Group	Number of isolates, n	IRR 2016–2024 (Reference: 2007–2015)	95% CI	p-value	aIRR 2016–2024 (Reference: 2007–2015)	95% CI	p-value
Staphylococcus aureus	9,163	1.06	1.01–1.10	0.009	1.10	1.05–1.14	< 0.001
Escherichia coli	5,321	1.05	0.99–1.10	0.11	1.04	0.98–1.10	0.16
Haemophilus/Moraxella spp.	5,062	0.81	0.76–0.85	< 0.001	0.81	0.76–0.85	< 0.001
Pseudomonas aeruginosa	4,899	1.00	0.94–1.05	0.90	0.97	0.91–1.02	0.24
Other Enterobacterales	4,571	1.05	0.99–1.11	0.12	1.03	0.97–1.10	0.27
Klebsiella pneumoniae	3,597	1.04	0.98–1.11	0.23	1.03	0.97–1.10	0.32
Enterobacter spp.	3,037	0.75	0.70–0.80	< 0.001	0.75	0.69–0.80	< 0.001
Serratia marcescens	2,754	1.15	1.06–1.24	< 0.001	1.14	1.06–1.23	< 0.001
Other *Klebsiella* spp.	2,529	1.28	1.19–1.39	< 0.001	1.29	1.19–1.39	< 0.001
Streptococcus pneumoniae	2,072	0.92	0.85–1.00	0.06	0.92	0.84–1.00	0.04
Stenotrophomonas maltophilia	1,741	1.12	1.02–1.24	0.01	1.11	1.01–1.22	0.03
Other non-fermenters	699	0.92	0.79–1.06	0.24	0.86	0.74–1.00	0.05
Acinetobacter spp.	554	0.79	0.67–0.93	0.005	0.80	0.68–0.94	0.009
Other *Streptococcus* spp.	375	1.16	0.95–1.43	0.15	1.24	1.01–1.52	0.04

aIRR: adjusted incidence rate ratio; IRR: incidence rate ratio

[1] Adjusted for covariates: sex, age group, geographic region, hospital type, hospital department, recovery method, sampling day after admission

Supplementary Table S4. Annual and period-based analysis of unadjusted and adjusted incidence rate ratios in pathogen distribution, sensitivity analysis, 2007–2024

Univariate Analysis						
Microorganism Group	IRR 2016–2024 (Reference: 2007–2015)	95% CI	p-value	Annual IRR	95% CI	p-value
Staphylococcus aureus	1.06	1.01–1.10	0.009	1.01	1.00–1.01	0.002
Escherichia coli	1.05	0.99–1.10	0.11	1.01	1.00–1.01	0.03
Haemophilus/Moraxella spp.	0.81	0.76–0.85	< 0.001	0.97	0.97–0.98	< 0.001
Pseudomonas aeruginosa	1.00	0.94–1.05	0.90	1.00	1.00–1.01	0.17
Other Enterobacterales	1.05	0.99–1.11	0.12	1.01	1.00–1.01	0.02
Klebsiella pneumoniae	1.04	0.98–1.11	0.23	1.00	1.00–1.01	0.34
Enterobacter spp.	0.75	0.70–0.80	< 0.001	0.97	0.96–0.97	< 0.001
Serratia marcescens	1.15	1.06–1.24	< 0.001	1.02	1.00–1.02	< 0.001
Other *Klebsiella* spp.	1.28	1.19–1.39	< 0.001	1.03	1.02–1.04	< 0.001
Streptococcus pneumoniae	0.92	0.85–1.00	0.06	1.00	0.99–1.00	0.27
Stenotrophomonas maltophilia	1.12	1.02–1.24	0.01	1.02	1.01–1.02	0.002
Other non-fermenters	0.92	0.79–1.06	0.24	0.98	0.96–0.99	0.005
Acinetobacter spp.	0.79	0.67–0.93	0.005	0.97	0.95–0.98	< 0.001
Other *Streptococcus* spp.	1.16	0.95–1.43	0.15	1.00	0.98–1.02	0.80

Multivariate Analysis [1]						
Microorganism Group	aIRR 2016–2024 (Reference: 2007–2015)	95% CI	p-value	Annual aIRR	95% CI	p-value
Staphylococcus aureus	1.10	1.05–1.14	< 0.001	1.01	1.01–1.01	< 0.001
Escherichia coli	1.04	0.98–1.10	0.16	1.01	1.00–1.01	0.05
Haemophilus/Moraxella spp.	0.81	0.76–0.85	< 0.001	0.97	0.97–0.98	< 0.001
Pseudomonas aeruginosa	0.97	0.91–1.02	0.24	1.00	0.99–1.01	0.99
Other Enterobacterales	1.03	0.97–1.10	0.27	1.01	1.00–1.01	0.06
Klebsiella pneumoniae	1.03	0.97–1.10	0.32	1.00	1.00–1.01	0.48
Enterobacter spp.	0.75	0.69–0.80	< 0.001	0.97	0.96–0.97	< 0.001
Serratia marcescens	1.14	1.06–1.23	< 0.001	1.02	1.01–1.02	< 0.001
Other *Klebsiella* spp.	1.29	1.19–1.39	< 0.001	1.03	1.02–1.04	< 0.001
Streptococcus pneumoniae	0.92	0.84–1.00	0.04	1.00	0.99–1.00	0.23
Stenotrophomonas maltophilia	1.11	1.01–1.22	0.03	1.01	1.00–1.02	0.006
Other non-fermenters	0.86	0.74–1.00	0.05	0.97	0.96–0.99	< 0.001
Acinetobacter spp.	0.80	0.68–0.94	0.009	0.97	0.96–0.99	< 0.001
Other *Streptococcus* spp.	1.24	1.01–1.52	0.04	1.01	0.99–1.03	0.38

aIRR: adjusted incidence rate ratio; IRR: incidence rate ratio

[1] Adjusted for covariates: sex, age group, geographic region, hospital type, hospital department, recovery method, sampling day after admission

Supplementary Table S5. Annual and period-based analysis of unadjusted and adjusted incidence rate ratio in resistance groups, sensitivity analysis, 2007–2024

Univariate Analysis				Multivariate Analysis [1]			
IRR 2016–2024 (Reference: 2007–2015)		Annual IRR		aIRR 2016–2024 (Reference: 2007–2015)		Annual aIRR	
IRR (95% CI)	p-value	IRR (95% CI)	p-value	aIRR (95% CI)	p-value	aIRR (95% CI)	p-value
Methicillin resistance within tested *Staphylococcus aureus* (601/9,151 = 6.6%)							
0.75 (0.64–0.88)	< 0.001	0.97 (0.96–0.99)	< 0.001	0.78 (0.66–0.91)	0.002	0.97 (0.96–0.99)	< 0.001
Third-generation cephalosporin resistance within tested Enterobacterales (3,268/21,759 = 15.0%)							
0.81 (0.76–0.87)	< 0.001	0.99 (0.99–1.00)	0.03	0.81 (0.75–0.87)	< 0.001	0.99 (0.99–1.00)	0.04
Carbapenem resistance within tested Enterobacterales (253/21,777 = 1.2%)							
3.21 (2.40–4.38)	< 0.001	1.14 (1.11–1.17)	< 0.001	3.27 (2.43–4.46)	< 0.001	1.14 (1.11–1.18)	< 0.001
Carbapenem resistance within tested *Pseudomonas aeruginosa* (987/4,890 = 20.2%)							
2.62 (2.27–3.02)	< 0.001	1.10 (1.09–1.12)	< 0.001	2.68 (2.33–3.10)	< 0.001	1.11 (1.10–1.13)	< 0.001
Carbapenem resistance within tested *Acinetobacter* spp. (80/551 = 14.5%)							
2.11 (1.34–3.38)	0.002	1.10 (1.05–1.15)	< 0.001	1.82 (1.15–2.95)	0.01	1.08 (1.04–1.13)	< 0.001

aIRR: adjusted incidence rate ratio; IRR: incidence rate ratio

[1] Adjusted for covariates: sex, age group, geographic region, hospital type, hospital department, recovery method, sampling day after admission

Supplementary Table S6. Full model results of adjusted incidence rate ratios across resistance groups, 2007–2024

Periods		Covariates: sex, age group, geographic region, hospital type, hospital department, recovery method, sampling day after admission													
aIRR 2016–2024<br>(Reference: 2007–2015)		Male sex<br>(Reference: female sex)		Age ≥ 60 years<br>(Reference: age < 60 years)		Central-West<br>East<br>North-East<br>North-West<br>West<br>(Reference: Central-East)		University hospital<br>(Reference: non-university hospital)		Non-ICU<br>(Reference: ICU)		Sputum<br>TBS<br>(Reference: BAL)		Day 5–10<br>Day 11–30<br>(Reference: Day 2–4)	
aIRR (95% CI)	p-value	aIRR (95% CI)	p-value	aIRR (95% CI)	p-value	aIRR (95% CI)	p-value	aIRR (95% CI)	p-value	aIRR (95% CI)	p-value	aIRR (95% CI)	p-value	aIRR (95% CI)	p-value
Methicillin resistance within tested *Staphylococcus aureus* (601/9,151 = 6.6%)															
0.78 (0.66–0.91)	0.002	1.05 (0.88–1.25)	0.62	1.54 (1.29–1.85)	< 0.001	2.34 (1.54–3.66)<br>0.79 (0.32–1.71)<br>1.52 (0.93–2.51)<br>1.85 (1.22–2.88)<br>4.68 (3.02–7.46)	< 0.001<br>0.58<br>0.10<br>0.005<br>< 0.001	0.95 (0.76–1.19)	0.65	1.05 (0.87–1.27)	0.60	0.98 (0.70–1.39)<br>0.70 (0.51–0.99)	0.89<br>0.04	1.33 (1.08–1.63)<br>1.82 (1.50–2.20)	0.006<br>< 0.001
Third-generation cephalosporin resistance within tested Enterobacterales (3,268/21,759 = 15.0%)															
0.81 (0.75–0.87)	< 0.001	0.91 (0.85–0.98)	0.02	1.23 (1.13–1.34)	< 0.001	0.75 (0.65–0.87)<br>0.35 (0.27–0.45)<br>1.54 (1.34–1.76)<br>0.59 (0.52–0.68)<br>1.29 (1.11–1.50)	< 0.001<br>< 0.001<br>< 0.001<br>< 0.001<br>0.001	0.99 (0.90–1.10)	0.91	0.87 (0.80–0.94)	< 0.001	0.88 (0.74–1.05)<br>0.88 (0.75–1.05)	0.15<br>0.14	1.34 (1.23–1.46)<br>1.55 (1.42–1.69)	< 0.001<br>< 0.001
Carbapenem resistance within tested Enterobacterales (253/21,777 = 1.2%)															
3.27 (2.43–4.46)	< 0.001	1.00 (0.76–1.33)	1.00	1.16 (0.87–1.58)	0.31	0.81 (0.49–1.36)<br>1.41 (0.81–2.45)<br>1.18 (0.72–1.94)<br>0.64 (0.40–1.04)<br>0.59 (0.33–1.05)	0.42<br>0.22<br>0.52<br>0.06<br>0.07	1.18 (0.84–1.66)	0.33	0.77 (0.58–1.02)	0.07	0.68 (0.40–1.25)<br>0.72 (0.43–1.28)	0.19<br>0.22	1.88 (1.36–2.63)<br>2.43 (1.75–3.40)	< 0.001<br>< 0.001

Carbapenem resistance within tested *Pseudomonas aeruginosa* (987/4,890 = 20.2%)															
2.68 (2.33–3.10)	< 0.001	1.11 (0.97–1.27)	0.14	0.96 (0.83–1.11)	0.54	0.41 (0.33–0.52)	< 0.001	0.94 (0.79–1.11)	0.44	1.19 (1.02–1.38)	0.02	0.76 (0.57–1.04)	0.08	1.06 (0.90–1.23)	0.50
						0.83 (0.61–1.11)	0.22					1.07 (0.80–1.45)	0.68	1.34 (1.15–1.56)	< 0.001
						0.97 (0.78–1.11)	0.76								
						0.35 (0.28–0.42)	< 0.001								
						0.33 (0.25–0.42)	< 0.001								
Carbapenem resistance within tested *Acinetobacter* spp. (80/551 = 14.5%)															
1.82 (1.15–2.95)	0.01	0.93 (0.59–1.49)	0.76	1.22 (0.74–2.04)	0.45	1.83 (0.63–6.09)	0.28	0.56 (0.31–1.00)	0.05	0.73 (0.42–1.25)	0.25	0.37 (0.12–1.62)	0.12	0.62 (0.35–1.06)	0.08
						4.92 (1.55–16.89)	0.007					0.55 (0.19–2.30)	0.32	0.44 (0.25–0.76)	0.003
						2.22 (0.85–6.91)	0.13								
						3.16 (1.26–9.60)	0.02								
						2.94 (0.95–10.26)	0.07								

aIRR: adjusted incidence rate ratio; BAL: bronchoalveolar lavage; ICU: intensive care unit; TBS: Tracheobronchial secretion
